# Protecting the child while preserving the relationship: Using baby’s relational withdrawal to gauge the effect of parental visitation

**DOI:** 10.1371/journal.pone.0196685

**Published:** 2018-05-03

**Authors:** Alexandra Deprez, Jaqueline Wendland, Line Brotnow, Arno C. Gutleb, Servane Contal, Antoine Guédeney

**Affiliations:** 1 Laboratory of psychopathology and health processes (LPPS), Institute of Psychology, University of Sorbonne Paris Cité, Paris, France; 2 Yale Child Study Center, Yale School of Medicine, New Haven, Connecticut, United States of America; 3 Environmental Research & Innovation (ERIN), Luxembourg Institute of Science and Technology (LIST), Belval, Luxembourg; 4 CESP Inserm U 1187, GHPVS Hospital Bichat Claude Bernard APHP, Paris, France; 5 University of Paris Diderot, Paris, France; TNO, NETHERLANDS

## Abstract

The impact of children’s interactions with parents in the context of out-of-home placements is receiving much-needed cross-disciplinary attention. However, the paucity of instruments that can reliably represent young children’s experiences of such interactions precludes a nuanced evaluation of their impact on wellbeing and development. In response to this empirical gap, the present study investigates children’s relational withdrawal as a clinically salient, easily observable and conceptually valid measure of infants’ and toddlers’ responses to parents. Relational withdrawal, challenging behaviors and salivary cortisol were assessed before, during and after parental visits. Conceptually, the findings suggest that observations of relational withdrawal correlate meaningfully with measure of neurobiological reactivity. Clinically, three profiles of cross-variable responses in children appeared, distinguishing between groups that experience increased, decreased or unchanged levels of stress in response to parental visits. Taken together, the findings lend empirical support to systematic observations of relational withdrawal to bolster evaluations of young children’s experience of parental visitation during out-of-home placements.

## Introduction

Child welfare services in Europe and North America continue to grapple with the controversies related to establishing and maintaining child-parent relationships when parental care has been temporarily or permanently suspended. Nowhere is this difficulty more pronounced than regarding parental visitation, prompting professionals to assess and balance children’s need for protection with that of preserving a relationship with primary caretakers.

At present, the dearth of developmentally sensitive empirical findings cause these decisions to be swayed by legal and social considerations. Indeed, although parental visitation appears at the epicenter of longstanding clinical and legal debates [1], specific effects on the development and wellbeing of the child, the biological parents and foster parents remain virtually unexplored in research literature [2].

Pursuant to the understanding of the detrimental impact of early toxic stress on long-term neurophysiological development, the importance of early intervention in the context of child abuse or neglect has received broad recognition in recent literature [3] However, the detection of such deleterious effects in young children is complicated by the lack of verbal language, limiting the arsenal of instruments gauging the child’s emotional reactions to observation-based evaluations. Substantial concerns have been raised about the validity of such assessments [4,5,6]. Indeed, most of the extant research is retrospective, relies on carer-reports and fails to account for the effect of factors such as child age and placement conditions [7,8,9]. From a clinical perspective, a majority of the available measures require a level of training and expertise that most childcare professionals in the field of infant and toddler mental health do not possess. In response to these issues, the present study explores the conceptual validity and clinical usefulness of an observational measure quantifying the immediate effects of parental visitation on children aged 6–36 months placed in institutional foster care.

## Measuring child responses to parental visitation

Child behavioral distress: In young, and especially pre-verbal children, direct effects of adverse events can be difficult to identify. Indeed, whereas older children and adolescents have cognitive and verbal capacities that can be more easily measured and interpreted, the assessment of infants and toddlers is generally restricted to behavioral responses. Experts in early child development have proposed a range of in-depth, observational instruments for trained professionals to gauge infant and toddler mental health and development. Clinical research instruments such as Brazelton’s Neonatal Behavioral Assessment Scale (NBAS, Brazelton & Nugent, 1995) and the Bayley Scales of Infant Development (BSID, Bayley, 1993) evaluate infant and toddler behavior and development on multiple dimensions, including somatic symptoms, affect and relational factors. However, these and other instruments are generally time-consuming and require specifically trained reporters. Furthermore, they are generally insensitive to changes occurring within the span of a few hours or a day, rendering them unhelpful with regards to detecting acute stressors.

Interactional patterns and relational withdrawal: In terms of young children’s behavioral responses, the concept of relational withdrawal—defined as ‘a *turn inward*, *to retract oneself as to defend*, *preserve one’s personality"*[10]—when persistent and/or intense is considered a relevant early marker of infant psychopathology and of maladaptive parent-child interactions. Relational withdrawal may reflect the baby’s immediate reactions to changing modalities of interaction or denote chronic distress due to mutual difficulties in adaptation over time [11].

On a micro-analytic level, transitory relational withdrawal is an essential and normative mechanism for regulating interactions in early childhood [12,13,14]. When faced with unusual, inadequate or unpleasant stimuli and interactions, babies show their distress by modulating their relational engagement. Thus, avoiding eye contact, not smiling, frowning, diverting attention away from people and toys as well as some self-regulatory and soothing activities can be understood as ways for the young child to communicate relational and/or physical discomfort [15,16,13, 17].

More clinically worrisome is the prolonged relational withdrawal that can be observed in the context of somatic illness, neurological and sensory processing disorders, severe forms of malnutrition, in non-organic failure to thrive, and chronic pain in infancy [18,19]. Such behaviors are also frequently observed in children presenting with separation anxiety, post-traumatic stress disorders, depression and autism spectrum disorders as well as in the context of child neglect [20, 14, 21, 22]. Prolonged relational withdrawal can also be a part of the child’s response to inadequate interactions pursuant to post-partum depression [23] and other parental mental health issues [24] As such, prolonged relational withdrawal can be considered a mutual adaptation disorder, partly resulting from and always affecting the quality of the child-parent interaction [25] Over time, chronic relational withdrawal correlates strongly with insecure attachment [20] and has been identified in conjunction with attachment disorders [26].

Thus, relational withdrawal could—for biological, developmental or relational reasons—be considered a marker of asynchronicity between the baby and its relational environment. Although it can be seen as adaptive when transitory (seconds or minutes), prolonged or systematic withdrawal from interpersonal interactions is a relevant indicator of maladaptation and predictive of future psychopathology [27].

Neurobiological reactivity in the context of child emotional development: Salivary cortisol is a neurobiological stress marker, reliably predictive of blood cortisol levels [28,29]. Given the non-invasive and inexpensive sampling procedure, it is currently one of the most frequently used objective indicators of stress in infants and young children [30, 31, 32, 33] Similarly to adults, the production of cortisol in children follows a predictable circadian rhythm from the age of two months. Under normal circumstances, the rhythm is characterized by peaks of cortisol thirty minutes after waking followed by a slow decline throughout the day [34]. A stressful event can be detected in the salivary cortisol concentration by a peak in concentration twenty to forty minutes after the incident. Such neurophysiologic stress-response can be triggered by social interactions and the development of the Hypothalamic-Pituitary-Adrenal (HPA) axis during the first years of life is lastingly impacted by the quality of early relationships [30]. Indeed, research has shown that HPA-axis abnormalities in early life predict an increased risk for psychopathology across the lifespan [35, 36, 37, 38]. In children exposed to environmental stressors, including disturbed, disrupted or abusive parent-child relationships, HPA-axis deviations range from hyper-activation and increased reactivity to the near-extinction of variability (no morning cortisol peak and a reduced mean concentration throughout the day) [39, 36, 37]. These findings have established the relevance of salivary cortisol in the context of child psychopathology and its psychosocial environment. Conversely, salivary cortisol levels can be used to qualify the effects of therapeutic interventions and this measure is thus broadly apt to inform clinical practices in the field of child psychopathology [40, 41, 42].

## The present study

In a sample of children aged 6–36 months temporarily removed from parental care, the present study aims to assess the links between relational withdrawal—conceptualized as a coping strategy and a reaction to the stress induced by caregiver interactions—and other indicators of child distress. More precisely, a decrease in relational withdrawal upon contact with a parent is viewed as a marker of the adequacy of this relational environment (influenced namely by caregiver sensitivity) as compared to the level of withdrawal demonstrated by the child before contact. We hypothesize the presence of distinct profiles of child reactions, present across measures of relational withdrawal, salivary cortisol level, and challenging behaviors.

### Method

#### Participants

15 children (8 males) aged 6–36 months (*M* = 17.0, *SD* = 6.9) living in a residential nursery setting due to temporary termination of parental care were recruited to participate in the study. The children all received regular visits from parents, at least one of whom demonstrated adequate French language fluency. Institutional foster care in France, Belgium and Luxemburg is offered in response to situations requiring the immediate suspension of parental custody. The average frequency of visits was once weekly and the visits occurred following a regular schedule. For this reason, the time elapsed since the last visit would have been approximately similar for each family. The institutions participating in the present study have all benefited from quality improvement schemes based on early childhood and infant development research including an decreased child/caregiver ratio, the presence of team psychologists, numerous staff training opportunities, organized parental visits and dedicated work on parent-child relationship. These units welcome a small number of children and emphasize the establishment of stable routines of care (including a dedicated reference person). The current study was conducted in one institution in Belgium and one in Luxemburg, both using the Loczy approach for the care of children [43].

#### Procedure

Children removed from parental care represent a vulnerable and difficult-to-access population. Accordingly, a cautious and careful recruitment process was established to protect of children, parents and carers. To this end, the study obtained unanimous, twice-renewed approval from the National Ethics Research Center in Luxembourg (CNR) and the Childhood National Ethics center in Belgium (ONE). Written informed consent from one or both parents and the appointed legal guardian were obtained in a multi-step process, allowing ample time and opportunity to withdraw from the study.

**Fig 1:**
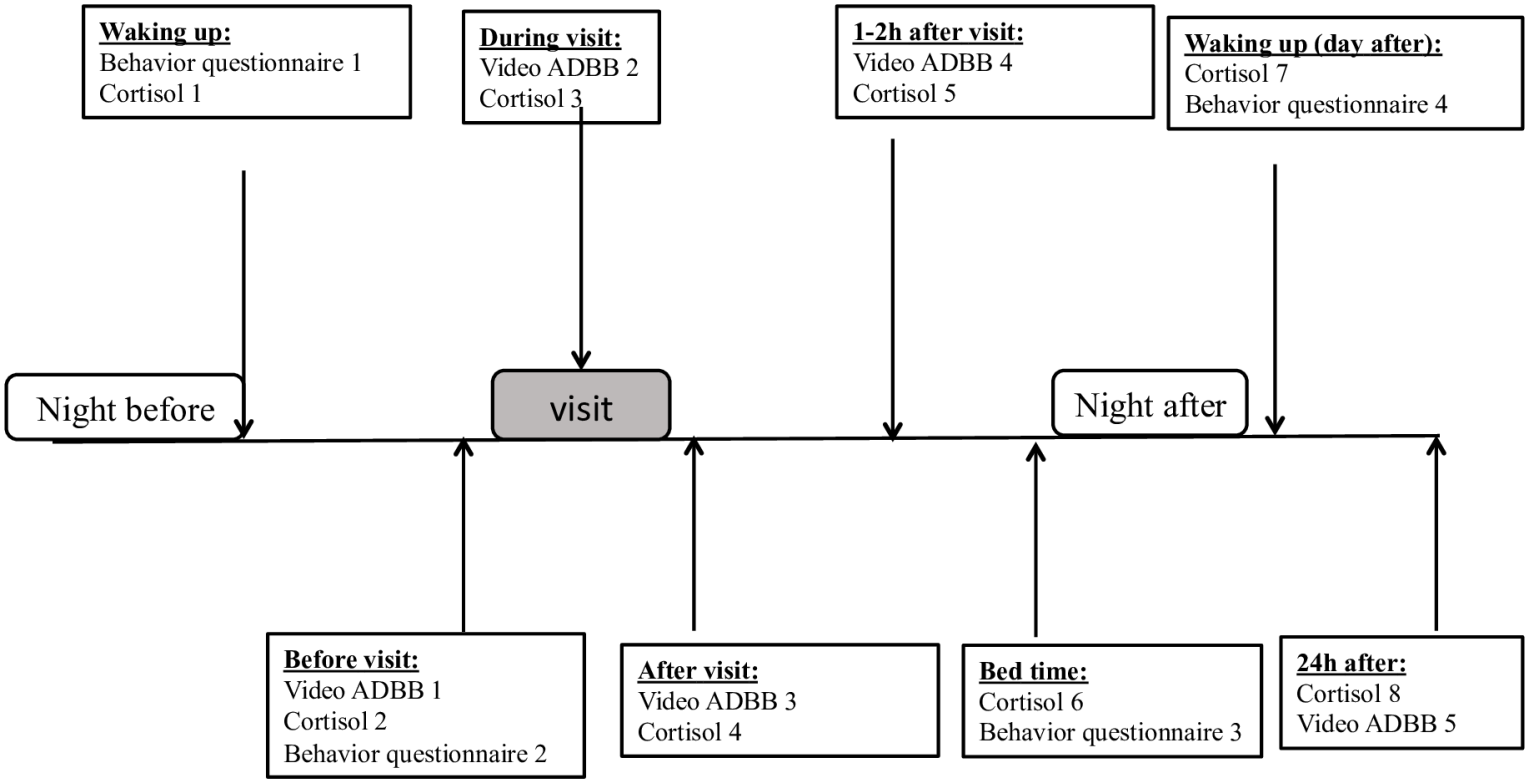
Data collection scheme.

Children were observed in their care institution on multiple occasions before, during, and after a planned visit from parents. Nurses completed questionnaires evaluating infants’ challenging behaviors on 4 occasions, at the end of both night and day shifts. Furthermore, infants’ interactions with nurses and parents were video recorded at 5 time points and coded by blinded evaluators with regards to establishing their level of relational withdrawal before, during and after the visit (see Fig 1). Salivary cortisol was collected at 8 time points. In order to ensure reliability, evaluations were conducted when the child had been fed and interrupted if the child showed signs of fatigue, discomfort or illness.

#### Measures

A multi-method approach was used to evaluate three dimensions of child reactions to parental visitation: child distress behaviors (day and night), relational withdrawal, and neurophysiological reactivity (salivary cortisol).

#### Relational withdrawal

The Alarm-Distress Scale (ADBB) [11] consists of eight items rated on a 5-point Likert scale ranging from 0 (normal behavior) to 4 (massively atypical behavior) and yielding a range of scores between 0–32. The eight items include facial expression, eye contact, activity level, self-stimulation gestures, vocalizations, delay in response to stimulation, relational quality, and attractiveness. The clinical cutoff score of 5 correlates with higher risk of developmental and psychopathological problems in the short and medium term [11, 14, 44, 45]. The ADBB scale has demonstrated adequate psychometric properties across cultural contexts [14] with regards to inter-rater correlations (r = 0.84), specificity (r = 0.78), and sensitivity (r = 0.82). A two-point difference in the overall ADBB score is considered clinically significant (Personal communication, Guedeney, 2010).

#### Salivary cortisol

Saliva samples were obtained using the Salimetrics Kit 8 times over the course of 24 hours. Concentrations were measured in micrograms per deciliter (ug/DL). In order to determine the baseline circadian production of cortisol, cortisol was measured on 3 occasions: 30 minutes after waking up and 30 minute before bed time the day of the visitation and 30 minutes after waking up on the day following the visitation. To establish the effect of the visit, cortisol was further measured on five occasions, immediately following each video-recorded interaction.

In order to obtain the samples, the child sucked or chewed on an absorbent cotton swab for 30–60 seconds. The child did not eat, drink or have their teeth brushed in the minutes preceding the sample. No salivation stimulator was used. The absorbent cotton was then stored in a sterile test tube provided for this purpose before being place in a freezer at -18 °C and preserved until they were transported to the laboratory for analysis.

#### Child distress behaviors

A systematic review of current literature did not identify a validated instrument evaluating challenging child behaviors that can be used in a 24 hour test-retest situation and that takes into account the child’s behavior during the day and at night. A behavior inventory was therefore constructed for the present study, based on items from the Symptom Checklist [46]. The behavioral inventory was created to take account of even slight changes in child nonverbal behavior over the course of a few hours. In addition to being conceptually rooted in the literature on normal and pathological child response to stressful events, this measure takes into account key aspects of babies’ life such as feeding, sleeping, somatic functioning, and non-verbal behavior. Furthermore, it was constructed to be user friendly, require no specific prior training, and be completed quickly (5–10 minutes).

Each item was rated in terms of absence/presence of the described behavior. Nurses who completed the inventory were those in charge of the child during the entire period of observation. They were instructed to interact with and observe the child as they ordinarily would and to rate the observed behavior at the end of their shift. Evaluating the child in their everyday context and integrating the assessment into nurses’ existing work responsibility was intended to increase the study’s ecological validity.

#### Data analytic strategy

The aim of the present study was to investigate the clinical value of relational withdrawal in terms of its ability to detect effects of parental visitation on young children. Based on videotaped interactions, subgroups of children were first identified based on the change in relational withdrawal observed upon initial contact with a parent. Then, the groups were compared with regards to salivary cortisol and challenging behaviors. Given the wide range of child age and duration of institutional placement, these variables were included as covariates. Analyses were performed using SPSS version 22.

### Results

#### Relational withdrawal

Based on changes in scores of relational withdrawal before and after parental visitation, three groups of children were identified: one showing decrease in relational withdrawal of 2 points or more on the ADBB upon contact with the parent (*n* = 4), another with a corresponding increase in relational withdrawal (*n* = 6), and a final group of children with no change in relational withdrawal (*n* = 5). See Table 1 for demographic information describing each group.

**Table 1:** Demographic information by reaction profile.

	Child sex	Mean child age in months (*SD*)	Mean duration of Placement in months (*SD*)	Mean level of change ADBB (*SD*)
Increase (*n* = 6)	66% male	12.8 (7.1)	9.7 (8.5)	4.6 (2.3)
No change (*n* = 5)	20% male	19.0 (11.9)	9.4 (5.6)	0.2 (0.8)
Decrease (*n* = 4)	75% male	21.1 (8.7)	12.8 (7.1)	-4.0 (1.6)

**Fig 2:**
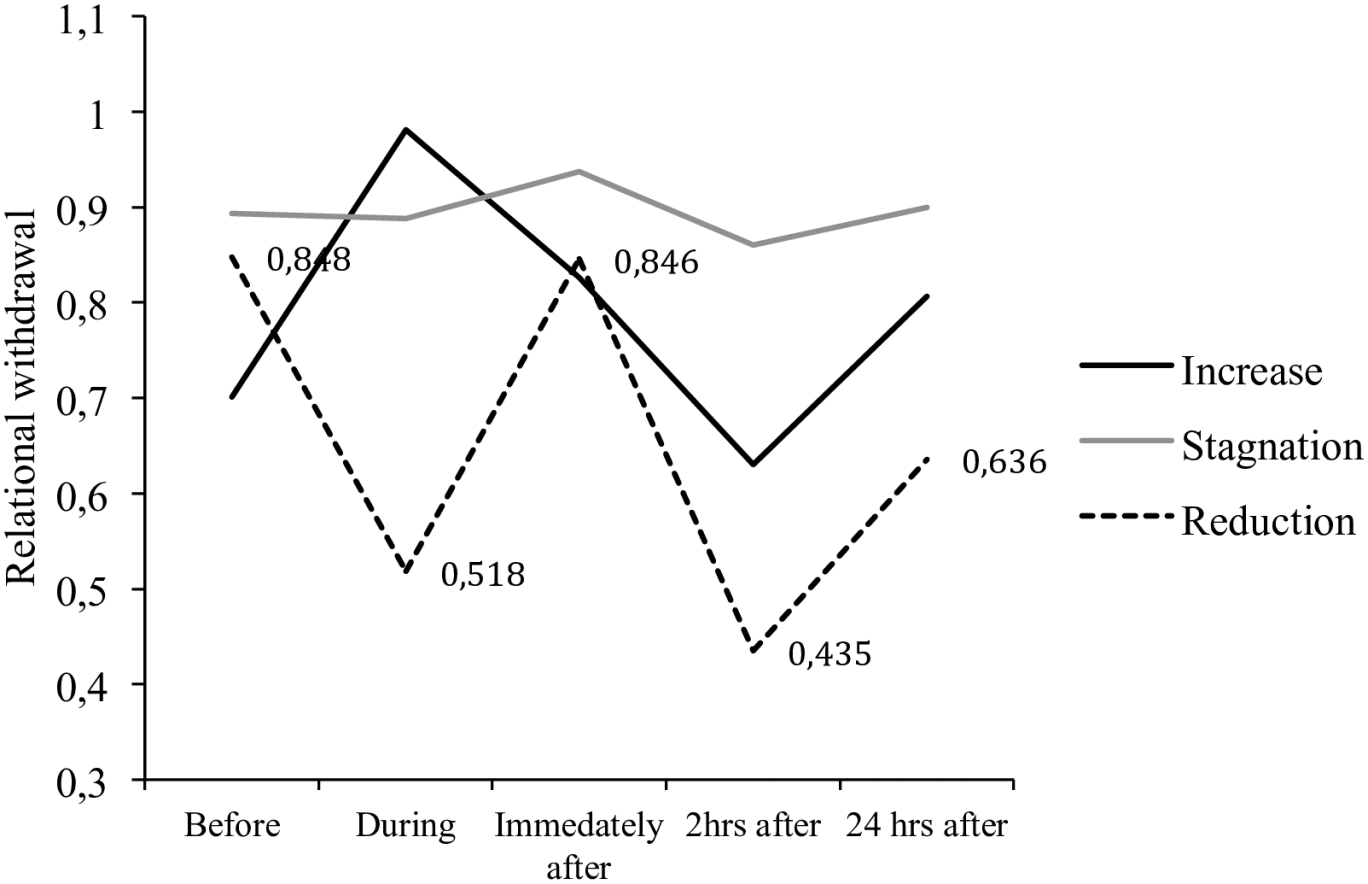
Estimated marginal means (logarithmic) for reaction profiles and repeated measures ADBB scores including age and duration of placement as covariates.

In order to examine differences in trajectories of relational withdrawal beyond the initial response to the parent(s), data were further analyzed using a mixed analysis of variance (ANOVA) with reaction profile as the between-subjects factor (decrease, increase or stagnation of relational withdrawal upon meeting parents) and the repeated measurement of ADBB as the within-subjects factor (5 time points). (See Fig 2).

To meet the assumption of variance homogeneity, data were transformed to logarithms, which satisfactorily normalized the distribution. Because Mauchly’s test indicated that the assumption of sphericity had been violated (*X*^*2*^_(9)_ = 20.97, *p* = .014), degrees of freedom were corrected using Huynh-Feldt estimates of sphericity (*ε* = .931).

The analysis revealed a significant and robust main effect of time (*F*_(3.73, 44.67)_ = 3.73, *p* = .001, *η*_*p*_^2^ = .346, *f* = .56) but not of group (*F*_(2, 12)_ = 1.49, *p* = .265, *η*_*p*_^2^ = 198, *f* = .5). However, a significant and similarly robust interaction effect was observed (*F*_(7.44, 44.67)_ = 4.05, *p* = .001, *η*_*p*_^2^ = .403, *f* = .66) suggesting that the three groups display different trajectories of relational withdrawal as a function of time. Thus, in addition to the difference observed during the child’s interaction with the parent(s), the groups distinguish themselves with regards to the trajectory of return to baseline levels of relational withdrawal. Importantly, these results remain significant after controlling for duration of placement and child age at evaluation and the effects remain in the large to very large range (*f* = .44-.74). Taken together, these findings suggest the presence of underlying profiles of relational withdrawal that can be meaningfully gleaned from the initial behavioral response to the parent, quantified by the ADBB scale.

#### Salivary cortisol

Following the initial empirical validation of the group categorization, salivary cortisol data were analyzed using a mixed-design ANOVA with reaction profile as the between-subjects factor (decrease, increase or stagnation of relational withdrawal upon meeting parents) and the repeated measurement of cortisol as the within-subjects factor (8 time points). To meet the assumption of variance homogeneity, outliers were Windsorized and missing data replaced with the mean sample score for the relevant time point. Less than 5% of data were missing in total and each child had a maximum of one altered score.

Because Mauchly’s test indicated a violation of the assumption of sphericity (*X*^*2*^_(27)_ = 52.44, *p* = .004), degrees of freedom were corrected using Huynh-Feldt estimates of sphericity (*ε* = .853).

In line with previous findings, the analysis revealed a significant main effect of time (*F*_(5.97, 71.69)_ = 6.06, *p* < .000, *η*_*p*_^2^ = .336, *f* = .54), indicating that, for the sample as a whole, mean levels of salivary cortisol differ during the course of a day. This remains true after controlling for duration of placement (*F*_(6.15, 67.64)_ = 2.34, *p* < .036, *η*_*p*_^2^ = .179, *f* = .39) but not when age at evaluation is included as a covariate (*F*_(5.99, 65.89)_ = 1.88, *p* < .097, *η*_*p*_^2^ = .146, *f* = .35).

**Fig 3:**
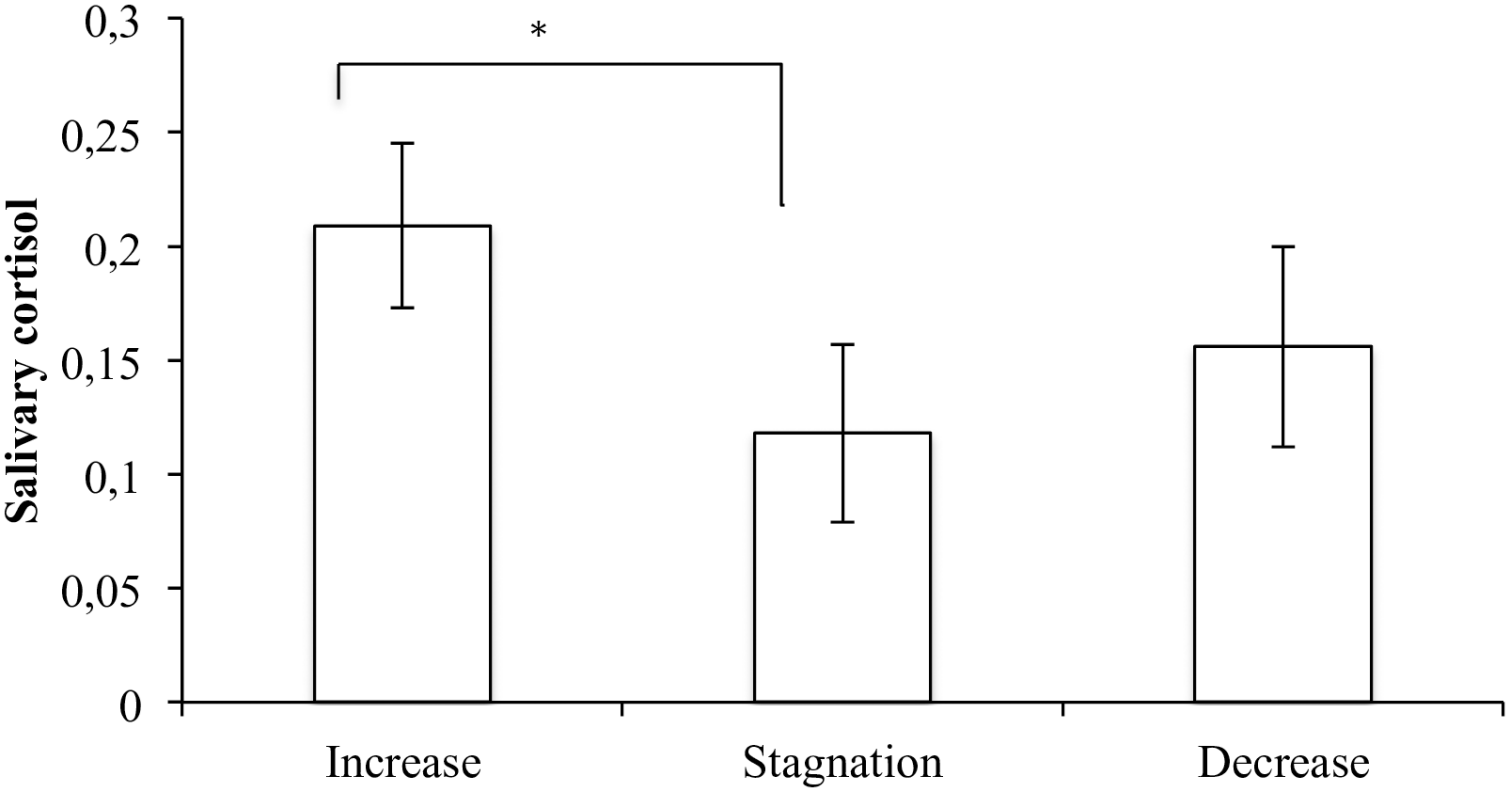
Estimated marginal means of salivary cortisol by group of relational withdrawal.

In line with hypotheses theorizing that relational withdrawal would be associated with neurophysiological reactivity, the analysis revealed a main effect of the reaction profile (*F*_(2, 12)_ = 7.22, *p* = .009, *η*_*p*_^2^ = .546, *f* = .98). The mean salivary cortisol level across all time points differed significantly between the three groups. Importantly, this pattern remained after controlling for both duration of placement (*F*_(2, 11)_ = 6.71, *p* = .012, *η*_*p*_^2^ = .550, *f* = .74) and child age (*F*_(2, 11)_ = 5.62, *p* = .021, *η*_*p*_^2^ = .505, *f* = .71). Specifically, post hoc analysis revealed that the group demonstrating increased relational withdrawal during the visit has statistically significantly higher mean levels of salivary cortisol than the stagnation group after controlling for multiple comparisons (Tukey HSD, *p* = .007). (See Fig 3).

**Fig 4:**
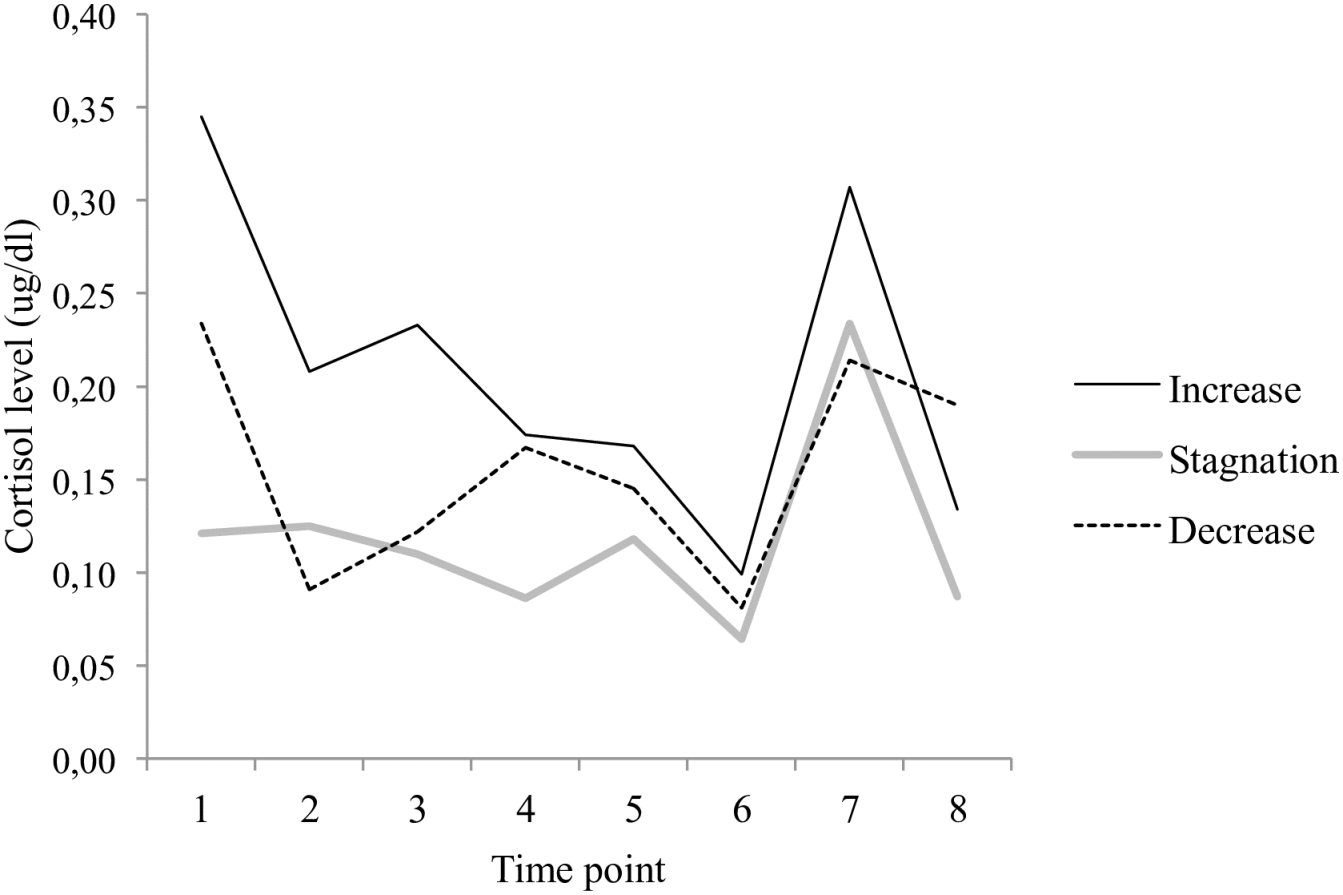
Estimated marginal means of salivary cortisol as a function of reaction profile (interaction).

No statistically significant interaction between reaction profile and cortisol levels was revealed (*F*_(11.95, 71.67)_ = 1.22, *p* = .290, *η*_*p*_^2^ = .168, *f* = .34). However, given the likely impact of the sample size sample on the statistical power associated with this analysis (as indicated by the presence of a medium sized, yet not statistically significant effect) and for the sake of future replication efforts, the below graph is included to show the trends observed in the present sample (see Fig 4).

Overall, the different profiles previously observed in terms of relational withdrawal thus also appear to be distinguishable in terms of HPA axis reactivity.

#### Child distress behavior index

Due to the high number of zero observations, data could not be normalized. A Kruskal-Wallis H-test showed no statistically significant baseline differences in challenging behaviors between the three groups at any of the four time points.

## Discussion

The present study used the ADBB scale of relational withdrawal to gauge the effects of parental visit on babies temporarily placed out-of-home. Three distinct profiles appeared based on observed variation of relational withdrawal upon initial parental contact. These groups were found to differ significantly in relational withdrawal over the course of 24 hours and also in terms of salivary cortisol. Overall, the results indicate that relational withdrawal can meaningfully be used to identify babies’ experience of parental contact in the context of temporary suspension of custody.

The decrease group showed a reduction of relational withdrawal level upon contact with parents. These babies seemed to heighten their social engagement during the visit as compared with baseline levels. Upon returning from the visit, data showed an increase of relational withdrawal followed by steady improvement (reduction) over the following 24h. Children in this group demonstrated an increase in cortisol levels but only at the time of separation from their parents. Taken together, these results suggest a positive impact of contact with parents, which remained beyond the initial reaction of protest upon separation. For these children, the separation may thus constitute a more substantial stressor than the interaction with their parents in itself. Pending replication of the present findings, it may be necessary to adopt practices helping these children cope with the stress of the separation.

The increase group showed an elevation of relational withdrawal level upon contact with parents. Generally, children in this group had a baseline level of relational withdrawal below the clinical threshold and lower than the other two groups prior to the visit and they withdrew strongly upon contact with parents. After the visit, relational withdrawal scores returned to baseline, suggesting an absence of distress linked to the separation itself. Overall, this group showed higher average cortisol levels at all time-points than the two other groups, which may be suggestive of a more stressful early environment or a more reactive neurophysiological profile. Importantly, no decrease in salivary cortisol was observed during contact with parents, highlighting the absence of a soothing effect of the interaction. Taken together, these findings seem to indicate that, as least for some children, contact with parents can be a stressful event that would need to be understood, acknowledged and taken into consideration when planning visits so that babies can receive enough support to offset the impact of relational stress [2]. A number of factors related to the developmental history of the child (e.g. premature relational disruptions, absence of attachment relationships) and of the psychosocial environment (parent/caretaker conflict, stressful life experiences) may be relevant to understand this stress response. Future research should consider such factors specifically.

The last group showed no visible reaction to visitation in terms of relational withdrawal. On average, these children demonstrated more severe levels of relational withdrawal than the other two groups at all four time-points and showed very limited variation in relational withdrawal behavior over the course of 24 hours. Children in this group also had lower average salivary cortisol level at all time-points than the two other groups. These results could be indicative of HPA axis inhibition, resulting in lower reactivity [39, 36, 37, 47]. Indeed, the absence of any emotional reaction to contact with parents could be a sign of overall heightened distress for the baby and thus indicate elevated risk of future psychopathology [47, 27]. This finding is all the more worrying as this group is “emotionally silent”. Indeed, these babies are not difficult to deal with; they don’t protest (anymore) and overlooking their despair is likely even though they may be more in need of help than the other groups.

Taken together, these sometimes counterintuitive findings highlight the potential for misinterpreting babies’ behaviors in the context of parental visits and thus for misunderstanding the kind of support and protection that could be needed to foster healthy development. The present study also highlights the need for more research to understand witch factors are affecting babies’ reaction to contact with parents. For example, the improvement in relational withdrawal during the visit coupled with the spike in cortisol during separation for the decrease group indicates a potential risk to social and emotional development not from the visitation but from recurring separations. Contrastingly, for the increase group, the visit with the parent seems to be the stressor and the lack of meaningful observable reaction at separation lends empirical support to this interpretation. Finally, the stagnation group appears unbothered although, in reality, may present with the most substantial need for support and protection considering they display higher level of sustained relational withdrawal overall.

The findings reported in the present study should be interpreted with a number of limitations in mind. First of all, the size of the sample severely limits statistical power and thus the complexity of the analyses that can be carried out. More complex, multilevel modeling will be needed to further elucidate and corroborate the present findings. Another limitation concerns the challenging behavior inventory. Although it is closely associated with the Symptom Checklist [46] the measure was created for the purposes of the study and had not been the subject to independent empirical validation. It is possible that such tool, to be effective, would require substantially more training. Furthermore, previous literature has indicated that the ADBB partly reflects child temperament[48], which could not be verified in the present study. Further studies should therefore include measures of both child temperament and mood. Finally, although the specific context of the present study—institutional foster care—allows for a more controlled environment, it has specific characteristics that limit direct comparison with other forms of out-of-home placements. Further studies on children cared for in foster families or in joint-custody contexts are needed to replicate the present findings. As outlined, such designs would however have to resolve the substantial challenges related to standardizing the data collection.

These limitations notwithstanding, the present findings provide initial support for the usefulness of relational withdrawal as a concept to understand babies’ point of view in challenging interpersonal situations, and in distinguishing between ***possible*** groups of young children presenting with distinct needs in response to parental visitation. Importantly, the results indicate that emotional responses following parental visitation may be substantially misrepresenting the young child’s experience. This highlights the need for systematic training of professionals to conduct careful observation and assessment of children’s levels of relational withdrawal. Clinically, understanding babies’ responses can help professionals monitor their adaptation to the situation and, if necessary, take very early therapeutic steps to promote sensitive interactions. Further observation-based research is needed to substantiate these findings.

In terms of child welfare services, the present findings provide empirical support in response to the controversies that are currently re-emerging concerning the effect of parental visits on children placed out of home [49, 6, 50, 51, 2]. This study suggests that the question to ask is not whether there is a benefit to parental contact for babies placed away from home, but for which babies the visit is most beneficial. In order to construct sensitive and ecologically feasible interventions to address this issue, future studies are needed to understand the interplay of the specific characteristics of the parent-child interactions, of the placement and of the child. In-depth analysis of the specific parenting skills (sensitivity, reflective function) likely to affect levels of relational withdrawal in the child are particularly susceptible of becoming targets for intervention. Understanding these factors could then contribute to developing decision making models in child protection, guaranteeing the maximum level of contact between babies and parents with minimum adverse effects on the developing child.

## Supporting information

S1 Minimal Dataset (XLSX)
